# Effects of Metronidazole as an Adjunct to Non-Surgical Periodontal Therapy on Insulin Resistance in Type 2 Diabetics

**DOI:** 10.3390/antibiotics10111400

**Published:** 2021-11-15

**Authors:** Ambrina Qureshi, Zeba Haque, Hina Qureshi, Waqas Ahmed Farooqui

**Affiliations:** 1 Department of Community & Preventive Dentistry, Dow University of Health Sciences, Karachi 74200, Pakistan; 2 Department of Biochemistry, Dow International Medical College, Dow University of Health Sciences, Karachi 74200, Pakistan; z.haque@duhs.edu.pk; 3 Department of Pathology, The Kidney Center Postgraduate Institute, Karachi 75260, Pakistan; dr.hinaqureshi02@gmail.com; 4 School of Public Health, Dow University of Health Sciences, Karachi 74200, Pakistan; waqas.ahmed@duhs.edu.pk

**Keywords:** non-surgical periodontal therapy, insulin resistance, antibiotics, type 2 diabetes mellitus, clinical trial

## Abstract

Treating periodontitis with metronidazole (MET) as an adjunct to scaling root planing (SRP) is suggested to have inconsistent effects on insulin resistance (IR) in type 2 diabetes mellitus (T2DM). This paper will present the effects of MET, in addition to SRP, on the homeostatis model assessment of IR (HOMA-IR). A three-arm clinical trial was conducted and analyses were performed on T2DM participants with periodontitis (*n* = 74) who completed follow-up visits at 3 and 6 months after the intervention. The observed between-group and within-group mean changes in IR were found using ANOVA with repeated measures, followed by a post-hoc analysis, and a *p*-value of ≤0.05 was considered significant. Between-group analyses showed no difference in the HOMA-IR at 3 months, but at 6 months the difference was significant (*p* = 0.046). Within-group analyses showed that the HOMA-IR was significantly reduced in both test groups (*p* ≤ 0.05) over the period of time. Adjunct use of MET may result in a sudden short-term lowering of the HOMA-IR level within 3 months that may not be retained over 6 months when compared to the sustained lowering of the HOMA-IR levels in T2DM when intervened with SRP without MET.

## 1. Introduction

Uncontrolled type 2 diabetes mellitus (T2DM) with raised levels of glucose is represented by higher levels of HbA1c. In normal conditions, the hormone called insulin, produced by the human pancreas, helps to utilize this glucose in the body. However, in an uncontrolled condition, the patients suffer from insulin resistance (IR) that does not allow cells to absorb and utilize glucose, thereby causing increased glucose levels in the body. Insulin resistance is commonly initiated due to a reduced sensitivity to insulin-mediated glucose clearance and an inability to produce glucose by liver enzymes [1]. Other than T2DM, IR is commonly found in older ages and in overweight, middle-aged individuals who are sedentary [2]. Insulin resistance is also said to be established as a result of low-grade inflammation [3,4] that further deteriorates the systemic functions and eventually increases the risk of T2DM [5]. It is theorized that the persistent Gram-negative bacterial challenge and host-mediated release of pro-inflammatory cytokines may have consequences extending beyond the periodontal tissues, leading to increased IR and poor glycemic control [6].

The homeostasis model assessment of IR (HOMA-IR) is a surrogate index of IR based upon the relationship between fasting glucose and insulin levels proposed by Matthews in 1985. It has been used as proxy assessment of insulin resistance (IR) [7] and is a good index of the balance between endogenous glucose production and cell insulin secretion, based on the Matthews formula [6]. In the general population, a normal-weight healthy individual aged <35 years has a HOMA-IR of 1.0 [8].

Studies have shown an increased level of IR as a result of progressive periodontitis in adult population groups such as in Korean [9], American [10], French [11], Finnish [12], and British [13] groups. However, conflicting results have been observed in other studies conducted among non-diabetic Korean and Spanish population groups [5,14]. Similarly, a preliminary study conducted in Pakistan among T2DM patients showed no correlation between the HOMA-IR and periodontal measures, including periodontal pocket depths (PPD) and clinical attachment loss (CAL) [4,15]. The investigators suggested that this may be due to the small sample size. Concordant results were found in a study where the HOMA-IR did not correlate with any periodontal examination results [16].

Very few clinical trials are available that reported the effect of periodontal preventive methods on IR in T2DM patients [16,17,18,19]. The current trial is aimed at evaluating the effect of periodontal treatment, scaling and root planing (SRP), with and without metronidazole (MET) on glycemic control registered by clinicaltrials.gov (accessed on 6 September 2021) [20]. A paper has already been published that evaluated the effects of metronidazole (MET) on glycemic control with HbA1c levels as the primary outcome variable [21]. The current paper is an addition that intends to report the results of the same trial with respect to the HOMA-IR levels of T2DM patients after periodontal intervention. The null hypotheses of this paper were that there will be no difference in the HOMA-IR levels between the three trial arms and that there will be no difference within the trial arms over the period of time measured at 3 and 6 months.

## 2. Results

The mean age of all the recruited participants was 52.26 ± 7.58 (range = 36–65 years) with a formal education of 10.54 ± 5.05 years. The mean number of teeth present at baseline was 24.92 ± 3.08 and only 0.83 ± 1.63 teeth on average were indicated for extraction. On average, the periodontitis of all recruited participants was found with a pocket depth (mean PPD) of 3.48 ± 0.87 mm and a mean CAL of 3.87 ± 1.03. On average, the BOP was found in 26.12 ± 9.96% of all the examined teeth sites with a range of 7.14–50% of the examined teeth sites.

### 2.1. Baseline Associations with the HOMA-IR before Random Allocation

Table 1 presents the cross-sectional description of the study and its association with the HOMA-IR in all the included participants (*n* = 150) before random allocation. The participants were mostly male and married. More than half of the participants were overweight, almost half of them belonged to a lower standard of living, only around 5% were smokers, and almost 10% reported substance abuse, other than smoking. Thirty percent of participants were under diabetes management that included insulin, or both insulin and an oral hypoglycemic. Out of all the variables as per the selection criteria, diabetes management was found to be associated with increased levels of the HOMA-IR (*p* ≤ 0.05). In addition, increasing age was also found to be significantly associated with the increased levels of the HOMA-IR (*p* ≤ 0.05).

### 2.2. Mean Changes in the HOMA-IR in the Three Arms over Time

**Figure 1:**
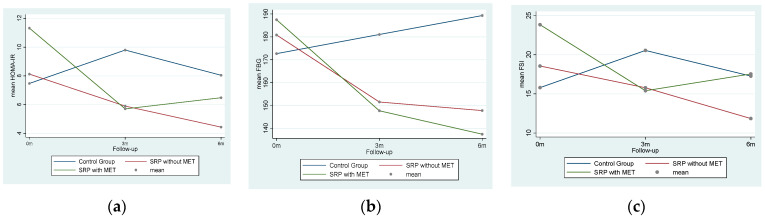
Profile plots of changes in mean HOMA-IR (**a**) over follow-up in three trial arms as a result of changes in FBG (**b**) and FSI (**c**).

Figure 1a–c shows the basic profile plots of changes in the mean HOMA-IR, FBG, and FSI over the follow-up from 0 months–3 months–6 months in the three trial arms. A drastic fall in IR levels was observed in the SRP with MET group within 3 months of the intervention, whereas a steady decrease in the HOMA-IR and FSI levels was seen in the SRP without MET group.

### 2.3. Intra-Group FSI and HOMA-IR Changes

With respect to the treatment arms, there was no statistical difference (*p* > 0.05) between the participants allocated to the three groups at baseline. This result is presented elsewhere [21]. The total number of participants who completed the 3-month follow-up was *n* = 97, and the total number of participants who completed the 6-month follow-up was *n* = 74, with *n* = 24 in the SRP with MET group, *n* = 26 in the SRP without MET group, and *n* = 24 in the control arms. Table 2 presents the intra-group mean differences in FSI and the HOMA-IR in T2DM patients from baseline to the 3- and 6-month post-intervention follow-up within each of the three trial arms. Significant reductions in FSI and HOMA-IR levels were observed in both test groups at the 3- and 6-month follow-ups. On the other hand, the FSI and HOMA-IR levels remained the same with no significant changes (*p* > 0.05). Since Mauchly’s test of sphericity showed that the variance in differences was not equal (*p* < 0.05), the *p*-values based on the Greenhouse–Geisser correction test results were reported.

### 2.4. Inter-Group FSI and HOMA-IR Changes

Table 3 shows the post-intervention between-group differences in the HOMA-IR at the 3-month and 6-month follow-ups. In addition to the HOMA-IR, between-group differences in FSI levels are also presented. There are no between-group differences in serum insulin levels between the test and control arms at the 3- or 6-month follow-ups (*p* > 0.05). On the other hand, the mean difference in the HOMA-IR levels between the three trial arms is significant at the 6-month follow-up but not at the 3-month follow-up. A post-hoc analysis shows that this difference is only significant in the SRP without MET group compared to the control arm (*p* = 0.036).

## 3. Discussion

The current trial results, compared to the HbA1c levels study that was previously published, allows a conclusion to be drawn, based on causality, that SRP with or without MET is equally effective in controlling the glycemic levels of T2DM patients [21]. Since the elevated pro-inflammatory markers in periodontal inflammation are suggested by the researchers to elevate IR in diabetics, there was a need to look into this change after T2DM patients were given antibiotics as an intervention [22]. The current results show that MET has no role in influencing the HOMA-IR in T2DM patients treated for periodontitis. A recently published narrative review on the use of Clindamycin in diabetics suggested that this antibiotic may be used as an alternative, and in addition to, SRP to treat severe progressive forms of periodontitis [23]. However, the authors did not show if Clindamycin had any effect on glycemic control in IR. Recently, a meta-analysis was performed that aimed to assess the effect of antibiotics on the HOMA-IR and also suggested that antibiotics did not significantly change the HOMA-IR [24]. This is in line with the results of the present trial. The reviewers further suggested that altering microbiota by any antibiotic may not have an effect on the metabolic status. However, the participants these reviewers were focusing on were those who were obese, with or without diabetes. An RCT by Rajkumar et al. found improved insulin sensitivity as a result of an intervention with probiotic supplementation, suggesting favorable effects [25]. Similarly, treating diabetics with Tinidazole (TNZ) and Ampicillin (AMP) for their periodontitis showed a significant improvement in IR [17] unlike the results of the current trial. This may be attributed to the effectiveness of TNZ, which is found to be almost two times more effective against anaerobic microorganisms in periodontitis than MET [4,26].

Although sudden falls in HOMA-IR levels were observed in the SRP with MET group over the period of 3 months, compared to the control group and the SRP without MET group, this fall was not statistically significant. On the other hand, the SRP without MET group showed a steady fall in HOMA-IR levels over the period of 6 months, which was also found to be significantly reduced compared to other trial arms. It is pertinent to mention here that FSI reduction was observed at the 3-month follow-up in both test groups, which was marginally significant (*p* = 0.04). The famous DPTT (Diabetes and Periodontal Therapy Trial), which was a multicentric trial with a larger number of samples treated with SRP without antibiotics, did not show any significant change in glycemic variables, including the HOMA-IR [19]. The DPTT investigators suggested that the latest clinical guidelines for diabetes did not support periodontal therapy as a means for glycemic control [27]. Similarly, Nishioka et al. also did not find any significant effect on IR as a result of periodontal therapy in their crossover trial where participants with borderline diabetes were treated with SRP [18]. On the other hand, Katagiri et al. advocated the use of antibiotics, particularly topical antibiotics such as minocycline ointment, in addition to SRP. They added that such an anti-infection therapy improves not only the glycemic control but also the BMI and IR [28]. An increased BMI is considered to be a major source of increased inflammation and a confounding factor between periodontitis and diabetes mellitus [29]. On the contrary, our baseline results before allocation did not find an association between BMI and IR, despite the fact that more than 50% of the selected participants were obese.

Although the idea was to recruit larger numbers of participants for the trial to assess the primary outcome that is HbA1c, there was a limitation in retaining this larger number at the 3-month and 6-month follow-ups. This limitation was due to the reason that our trial participants were unable to turn up for their follow-up visits in a fasting state, which was required to assess blood glucose and serum insulin levels as per protocol [21]. This resulted in compromising the sample size. Furthermore, although the increasing age of the participants was directly associated with the increased levels of the HOMA-IR before allocation, the trial participants were randomly allocated into three groups. This limitation was, therefore, overcome with respect to selection bias. Another limitation of the study was that no systematic data was collected pertinent to side effects as a result of the usage of the antibiotic (MET) by the participants in one of the three arms. However, we assumed that there were no side effects, since none of the participants reported any side effects on their own. Similarly, there was no recording of the compliance of the participants with respect to oral hygiene maintenance, so it is difficult to tell whether the participants were really following the instructions or not. In any case, keeping oral hygiene is important not only in normal situations, but also in conditions where the oral microbial burden could be high, such as in diabetes. Further studies may be targeted towards understanding the role of probiotic-based toothpastes in the routine management of oral hygiene in diabetics like those studied previously in healthy adult populations [30].

## 4. Materials and Methods

A single blind three-arm clinical trial was conducted in the tertiary care healthcare center of Karachi. The trial was registered at clinicaltrials.gov (NCT 03343366) and was conducted in line with the CONSORT guidelines [20,31]. The study flow chart is published elsewhere [21]. The ethical approval was sought by the institute (IRB-900/DUHS/Approval/2017/146) and the detailed methodology, along with the description of the target population, is presented elsewhere [21]. The sample size of *n* = 150, with *n* = 50 in each group, was calculated considering HbA1c levels as the primary outcome variable; its details can be found elsewhere [21]. The sample size was calculated using a 0.7%, 0.6%, and 0.06% reduction in HbA1c levels after a 3-month intervention in three groups: the SRP and antibiotic group, the SRP only group, and the no treatment group, respectively [32]. The total sample size calculated was *n* = 105, which was raised to a rounded number (*n* = 150) by adding a 40% dropout rate considering the fact that the trial was conducted in a developing country [33]. The selection criteria included T2DM patients managed with an oral hypoglycemic, insulin, or both, with HbA1c levels between 6.5% and <14%; the ages ranged between 35 and 65 years, and periodontitis was defined as ≥2 inter-proximal sites with ≥4 mm of CAL, which is the upper limit of Stage II and above as per newer classifications [34].

The types of interventions in three arms included SRP with MET, SRP without MET, and delayed treatment (DT) in the control group at the end of the 6-month trial. Oral hygiene instructions (OHI) were given to all the participants at the baseline. Full-mouth SRP in one sitting was provided through a combination of ultrasonic scaling on medium intensity and hand instrumentation using sharpened and sterilized curettes required to smoothen certain irregular areas of root surfaces [35]. Metronidazole (MET) 400 mg was added to the first test group thrice a day for 10 days [36]. All participants were instructed to brush their teeth using soft-bristled manual toothbrushes and fluoridated toothpaste twice daily (in the morning after breakfast and at night before sleeping) using a modified bass technique. Although previous studies have shown the improved efficacy of powered toothbrushes compared to manual toothbrushes [37], half of the participants were of a low living standard, so in order to have the same instructions for all the study participants, a similar brushing method using a manual toothbrush was recommended. This technique was demonstrated to them using a typodont model and toothbrush at baseline and at each follow-up as a reinforcement. The fasting serum insulin (FSI) levels were measured using the Abbott^®^ ARCHITECT i2000 System [38]. Fasting blood glucose (FBG) was tested using a standardized Accu Chek^®^ Guide device by Roche, and the results have already been presented previously [20]. Both these values were used to calculate the HOMA-IR as a proxy measure of insulin resistance, which was calculated using the online HOMA-IR calculator [39]. The periodontal measures assessed were bleeding on probing (BOP), PPD, and CAL using a UNC-15 periodontal probe in all teeth on six sites.

Stata v. 14.2 (StataCorp LLc, College Station, TX, USA) was used to describe the results. A Shapiro–Wilk test was used to check the normality of the dependent variable (HOMA-IR) considering a *p*-value of >0.05. Since the overall data with respect to the HOMA-IR level at baseline and follow-up was normally distributed (*p* > 0.05) in each arm, ANOVA with repeated measures was applied to observe mean changes. A post-hoc analysis was performed and a *p*-value of ≤0.05 was considered significant.

## 5. Conclusions

Adjunct use of MET may result in a sudden lowering of the HOMA-IR levels in 3 months within the group, but the reduction may not be retained over a longer period of 6 months, especially when compared to the sustained lowering of HOMA-IR levels as observed in the SRP without MET group. Therefore, the aim of prescribing MET may be dependent on the level of severity of the periodontitis, rather than as a requirement to improve glycemic control or insulin resistance in T2DM patients. However, considering the limitation of the study due to a compromised sample size, it is recommended that future studies be conducted with a larger number of participants, including T2DM participants with periodontitis.

## Figures and Tables

**Table 1:** Baseline characteristics (categorical) of participants before allocation [*n* = 150].

Variables	Categories	*n* (%)	HOMA-IR Coef. (*p*-Value *)
Gender	Female	68 (45.3)	--
	Male	82 (54.6)	−0.05 (0.962)
BMI Status	Underweight	5 (3.3)	--
	Normal	44 (29.3)	3.47 (0.292)
	Overweight	80 (53.3)	3.65 (0.255)
	Obese	21 (14)	1.80 (0.603)
Living Standard (*n* = 125)	Low	61 (48.8)	--
	Middle	35 (28)	1.30 (0.256)
	High	29 (23.2)	0.34 (0.775)
Smokers (*n* = 144)	No	136 (94.4)	--
	Yes	8 (5.5)	0.75 (0.756)
Substance Abuse (*n* = 144)	No	130 (90.2)	--
	Yes	14 (9.7)	0.79 (0.673)
Co-morbidities (*n* = 147)	None	80 (54.4)	--
	Hypertension	33 (22.4)	1.66 (0.255)
	CVD	9 (6.1)	1.21 (0.624)
	CVD + hypertension	4 (2.7)	−2.5 (0.489)
	Others	21 (14.2)	0.02 (0.990)
Diabetes management	Hypoglycemic	106 (70.6)	--
	Insulin	19 (12.6)	4.70 (0.006)
	Both	25 (16.6)	3.47 (0.021)
Regular Exercise	No	66 (44)	--
	Yes	84 (56)	−1.46 (0.200)
Healthy Diet	No	38 (25.3)	--
	Yes	112 (74.6)	0.98 (0.452)
Age (years)			0.14 (0.046)
Formal education (years)			0.09 (0.384)
Bleeding on probing (%)			0.00 (0.961)
Mean Periodontal pocket depth (mm)			0.90 (0.162)
Mean Clinical attachment loss (mm)			0.59 (0.277)

CVD = cardiovascular diseases. * Significant difference (*p* ≤ 0.05)

**Table 2:** Post-intervention within-group mean differences in glycemic variables.

Variables	SRP + MET + OHI	∆	*p*-Value *	SRP + OHI	∆	*p*-Value *	DT + OHI	∆	*p*-Value *
FSI (F0)	23.83 ± 16.41	--	0.041	18.55 ± 14.44	--	0.046	15.80 ± 9.80	--	0.107
FSI (F3)	15.40 ± 6.55	−8.43		15.77 ± 12.53	−2.78		20.55 ± 16.78	4.75	
FSI (F6)	17.53 ± 10.98	−6.3		11.85 ± 5.99	−6.7		17.26 ± 6.75	1.46	
	F-value = 4.97; MSE (df) = 49.0(29)			F-value = 4.48; MSE (df) = 23.75(32)			F-value = 2.51; MSE (df) = 46.85 (34)		
HOMA-IR (F0)	11.32 ± 9.72	--	0.022	8.12 ± 6.46	--	0.013	7.47 ± 7.21	--	0.070
HOMA-IR (F3)	5.70 ± 3.03	−5.62		5.89 ± 4.42	−2.23		9.79 ± 8.78	2.32	
HOMA-IR (F6)	6.47 ± 4.77	−4.85		4.43 ± 2.26	−3.69		8.04 ± 4.27	0.57	
	F-value = 5.63; MSE (df) = 24.08(29)			F-value = 6.87; MSE (df) = 9.25 (32)			F-value = 3.24; MSE (df) = 9.63 (34)		

FSI = Fasting Serum Insulin (mlU/L), F0 = baseline, F3 = 3 months, F6 = 6 months. ∆ = Intra-group mean differences between baseline and each follow-up reading. * Significant difference (*p* ≤ 0.05) and F-values measured by RMANOVA (based on Greenhouse–Geisser correction). MSE (df) = mean square error (degree of freedom).

**Table 3:** Short-term (3-month) and long-term (6-month) effects of SRP with MET and SRP without MET interventions on HOMA-IR.

	At 3-Months Follow-Up			At 6-Months Follow-Up		
Glycemic Variables v/s Intervention	∆	*p*-Value *	*p*-Value **	∆	*p*-Value *	*p*-Value **
FSI (mlU/L)						
∆ SRP + MET + OHI–DT + OHI	−5.14	0.371	0.437	0.26	0.108	0.995
∆ SRP + OHI–DT + OHI	−4.77		0.441	−5.41		0.163
∆ SRP + MET + OHI–SRP + OHI	−0.37		0.995	5.68		0.155
HOMA-IR						
∆ SRP + MET + OHI–DT + OHI	−4.08	0.081	0.130	−1.56	0.046	0.523
∆ SRP + OHI–DT + OHI	−3.89		0.119	−3.61		0.036
∆ SRP + MET + OHI–SRP + OHI	−0.19		0.995	2.04		0.346

∆ = mean difference. * Significant difference between three groups (*p* < 0.05). ** Significant difference at *p* ≤ 0.05 calculated through post-hoc Bonferroni test.

## Data Availability

The datasets generated and analyzed during the current study are not publicly available as it is part of PhD research and may be available from the corresponding author on reasonable request only after thesis public defense.
